# Species-specific genetic variation in response to deep-sea environmental variation amongst Vulnerable Marine Ecosystem indicator taxa

**DOI:** 10.1038/s41598-020-59210-0

**Published:** 2020-02-18

**Authors:** Cong Zeng, Ashley A. Rowden, Malcolm R. Clark, Jonathan P. A. Gardner

**Affiliations:** 1 College of Animal Science and Technology, Hunan Agricultural University, Changsha, China; 2 School of Biological Sciences, Victoria University of Wellington, Wellington, 6140 New Zealand; 3 Coasts and Oceans National Centre, National Institute for Water and Atmospheric Research, Private Bag, 14901 Kilbirnie Wellington, New Zealand

**Keywords:** Conservation biology, Marine biology

## Abstract

Understanding the ecological processes that shape spatial genetic patterns of population structure is critical for understanding evolutionary dynamics and defining significant evolutionary and management units in the deep sea. Here, the role of environmental factors (topographic, physico-chemical and biological) in shaping the population genetic structure of four deep-sea habitat-forming species (one sponge - *Poecillastra laminaris*, three corals - *Goniocorella dumosa*, *Madrepora oculata*, *Solenosmilia variabilis*) was investigated using seascape genetics. Genetic data (nuclear and mitochondrial sequences and microsatellite multilocus genotypes) and environmental variables were employed to build individual-based and population-level models. The results indicated that environmental factors affected genetic variation differently amongst the species, as well as at different geographic scales. For individual-based analyses, different environmental variables explained genetic variation in *P. laminaris* (dissolved oxygen), *G. dumosa* (dynamic topography), *M. oculata* (sea surface temperature and surface water primary productivity), and *S. variabilis* (tidal current speed). At the population level, factors related to current and food source explained the regional genetic structure in all four species, whilst at the geomorphic features level, factors related to food source and topography were most important. Environmental variation in these parameters may be acting as barriers to gene flow at different scales. This study highlights the utility of seascape genetic studies to better understand the processes shaping the genetic structure of organisms, and to identify environmental factors that can be used to locate sites for the protection of deep-sea Vulnerable Marine Ecosystems.

## Introduction

How spatially variable environmental and habitat features influence evolutionary processes and population genetic connectivity in the deep sea is poorly understood^1–3^. The deep-sea environment experiences increasing pressure, decreasing pH, and generally decreasing temperature, with increasing depth^4^. Such single factor or multi-factorial gradients may strongly influence dispersal, settlement and recruitment patterns of deep-sea taxa^5–7^. Additionally, features such as bottom currents^8^, surface currents^9^ and bathymetry may play important roles in shaping the patterns of genetic connectivity amongst populations in many deep-sea species^5,10,11^, with the result that multiple environmental factors may act as barriers to or promoters of gene flow in the deep sea^8,9,12^ and thereby strongly influence population genetic structure at different spatial and even temporal scales. However, the complexity of the deep-sea physico-chemical environment and the logistical difficulties of sampling (both biological specimens and environmental data) such a large biome have limited our ability to understand multispecies patterns of population genetic structure and how these are influenced by environmental variation^3,13–15^.

Deep-sea vulnerable marine ecosystems (VMEs) are easily disturbed by anthropogenic activities, are very slow to recover, or may never recover, and are physically or functionally fragile^16^. Coral reefs and sponge gardens are two VMEs in the deep sea^16^, indicator taxa of which include scleractinian coral species that build reef frameworks and large and/or abundant demo- or hexactinellid sponges that provide physical habitat for other organisms^17^. Although there are no studies that have quantified the relationships between environmental factors and the maintenance of genetic structure in the deep sea, there are several studies from other research areas that provide information on the importance of some environmental factors for coral reefs and sponge gardens. Generally, factors such as depth, temperature, salinity, particulate organic carbon (POC), and aragonite (for corals) and silica (for sponges) concentrations have been recognised as influencing the formation of deep-sea coral and sponge habitats^18–25^. In addition, topography and sediment supply are also regarded as important factors influencing the distribution of coral and sponge habitat-forming deep-sea species^26,27^. At small spatial scales, internal tidal mixing and pH variation may have significant effects on coral (and presumably sponge) larvae and their ability to connect different populations^28,29^. However, the relative influence (weak versus strong) and the mechanism of action by which these environmental variables moderate the interaction between habitat and population genetic structure and connectivity are largely unknown.

The discipline of seascape genetics is extended from landscape genetics^30,31^ and attempts to explicitly integrate spatial ecological information with population genetic data to characterise marine environmental factors that contribute to genetic connectivity and/or population genetic isolation^32,33^. Selkoe *et al*. summarised seascape genetics studies over the last 10 years and found that temperature, oceanography (e.g., currents) and geography (e.g., distance) showed equal prevalence of influence on spatial genetic patterns of shallow water species^34^. In addition, the review revealed that > 20 other factors also influence connectivity (gene flow) at distinct spatio-temporal scales. This latter point highlights the complexity of seascape genetics, in particular for the deep sea for which far less is known than in shallow/coastal waters, and there is high uncertainty about species-specific responses at different scales in time and in space. Thus, whilst seascape genetics holds much promise for delivering a new understanding of how environmental variation influences genetic variation (e.g., seascape genetics can help to identify significant spatial differentiation even when the genetic signal is weak^35,36^) it is important to appreciate that seascape genetics is in its infancy and we are still learning about its limitations and possibilities. This is particularly the case in the deep sea, where there is an information vacuum for the largest single biome on the planet^13^.

Within the New Zealand Exclusive Economic Zone (EEZ), many species are endemic and most have distributions that encompass a range of environments, and are therefore amenable to quantification of the effect of environmental variation on population genetic variation and genetic connectivity^37,38^. Species-specific genetic variation in VME indicator taxa in the New Zealand region may be caused by a number of different factors, including historical processes (phylogeography), connectivity (high versus low gene flow within and amongst populations), and environmental variation (contemporary selection pressure)^3,14^. Whilst there is, at present, no indication that the patterns of species-specific genetic structuring are attributable to local or regional adaptation, the recognition of its existence as a pre-existing condition for seascape genetics analyses is important. Typically, genetic differentiation is described using neutral markers^33,39^, but with new marker types and new analytical procedures there is increasing focus on non-neutral markers (genes or non-coding regions closely linked to genes), at least some of which may be of particular value in determining the cause and functional mechanism of pronounced patterns of genetic structure that may arise as a consequence of selection^15,34,37,40,41^.

**Figure 1:**
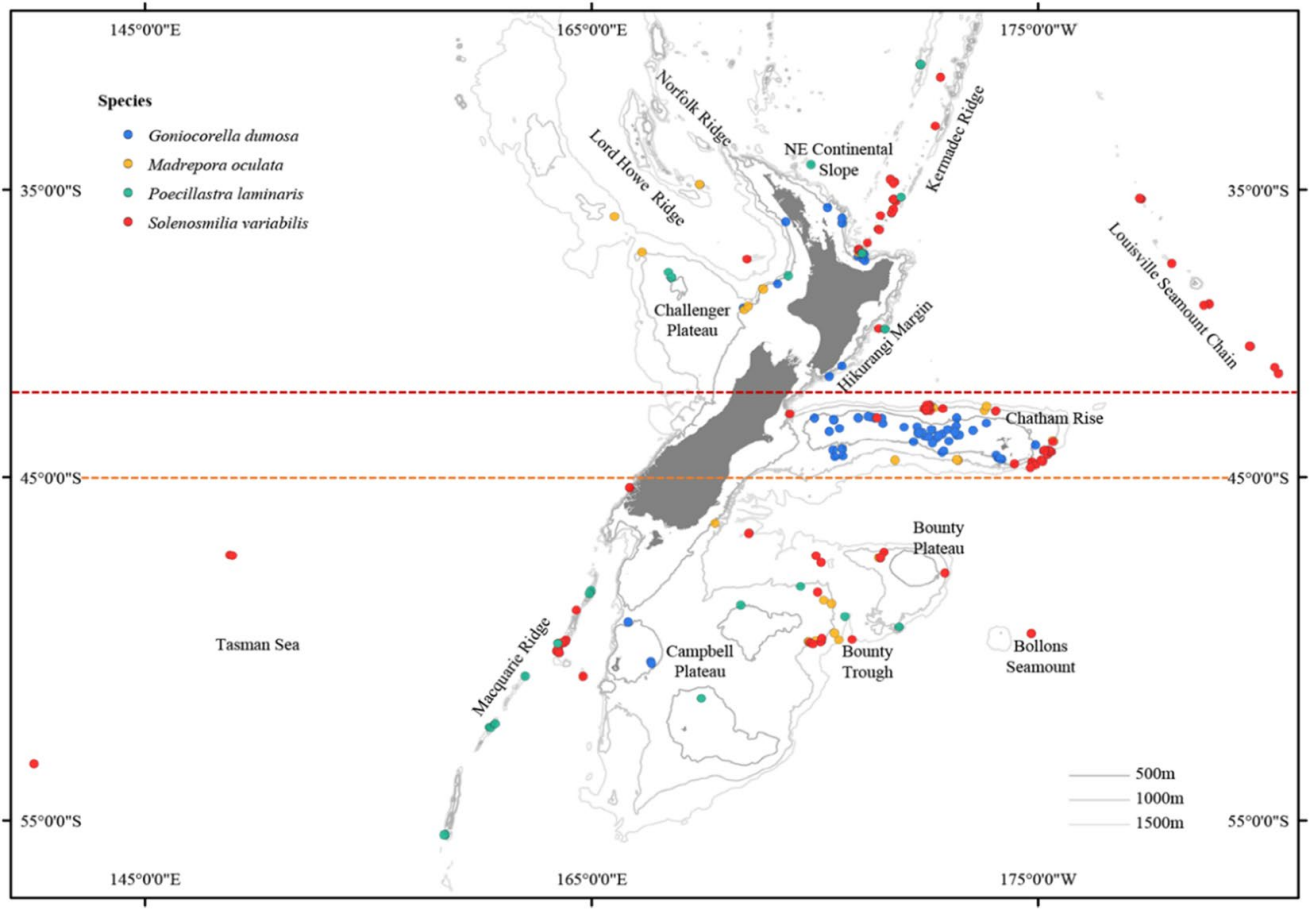
Map showing the distribution of samples amongst lower bathyal biogeographic provinces (yellow dashed line is the boundary between the northern and southern provinces), regions (yellow and red dashed lines indicate the boundaries for the north-central-south regions), and geomorphic features (named features) used for the analysis of genetic population structure for three species of deep-sea stony corals (blue, yellow and red dots) and one species of demosponge (green dots).

Because of its highly variable seafloor topography and bathymetry and its complex patterns of oceanic dynamics, New Zealand presents an ideal opportunity to test the effect of multi-factorial environmental influences on the genetic variation of deep-sea species. Such testing may be carried out at different spatial scales that are relevant to species’ distributions across the New Zealand region. Firstly, at the largest spatial extent, global assessment of the biogeography of the deep sea based on depth, temperature, salinity, dissolved oxygen, particulate organic carbon (POC), hydrographic and bathymetric parameters^42^ indicates that the New Zealand region is composed of two provinces, a northern (BY6) and a southern (BY10) province with a boundary at latitude ~45°S. Secondly, superimposed on top of this biogeographic structure are three major currents and their associated fronts around New Zealand - the Tasman Front, the Subtropical Front and the Subantarctic Front^43^. These currents and frontal systems differentiate water masses in the north and the south of the region, and also influence the direction of northern and southern currents across the Chatham Rise (to the east of New Zealand) that may reduce or perhaps even block gene flow across this feature. Corresponding to these potential hydrographic boundaries, three distinct regions have been recognised^3^ - above 42°S is the north region, between 42–45°S is the central region that contains the Chatham Rise, and below 45°S is the south region. Thirdly, the seafloor of the New Zealand EEZ is distinguished by different geomorphic features (such as seamounts, plateaux and ridges) that are characterised by different topographies and hydrodynamics that may influence genetic differentiation^44,45^. As such, populations on major geomorphic features (e.g., the Kermadec Ridge, the Chatham Rise, the Campbell Plateau – see Fig. 1) may be genetically isolated one from another, and this situation may therefore give rise to species-specific patterns of spatially explicit population genetic structure^3,14^. For example, *Poecillastra laminaris* (demosponge) and *Goniocorella dumosa*, *Madrepora oculata* and *Solenosmilia variabilis* (stony corals) are all common VME indicator taxa that are widely distributed throughout the New Zealand EEZ (Fig. 1). Previous studies have shown that different patterns of genetic structure are observed amongst these four species despite large overlaps in their distributions. Significant genetic differentiation was detected at the bioprovince, regional and geomorphic features scales in both *P. laminaris* and *S. variabilis*, at the regional and geomorphic features scales in *G. dumosa*, but only at the regional scale in *M. oculata*^3,14^. These different patterns of genetic structure may be related, at least in part, to species-specific reproductive strategies^1,6^, but a major limitation of research of this kind is that we know very little about the mode of reproduction or the larval dispersal ranges of most deep-sea species^1^. Whilst no studies have determined the reproductive strategy or mode of larval dispersal of the sponge, *P. laminaris*, deep-sea sponges are expected to produce lecithotrophic larvae with limited dispersal capacity^14^. The three coral species are expected to have very similar modes of reproduction and of larval dispersal^46–49^, are expected to be seasonal gonochoric spawners (although *S. variabilis* may be a continuous spawner^7^) with relatively short planktonic dispersal phases of perhaps a few days or weeks^7,50^. There is an indication that larval dispersal potential may be positively correlated with oocyte size amongst *Goniocorella dumosa*, *Madrepora oculata* and *Solenosmilia variabilis*^3^.

To explore how environmental variation may affect patterns of genetic structure, in the present study, environmental variables and genetic data were employed to quantify the relationships between environmental and genetic variation of these four VME indicator taxa at three different spatial scales. Three main hypotheses were tested: (1) that species-specific genetic variation is independent of environmental variation at the level of individuals within the New Zealand region, and for populations at different spatial scales, (2) that factors such as variation in silicate (for the sponge) and calcite (for the corals) explained significant variation in species-specific genetic variation, and (3) that multispecies genetic variation is associated with a common suite of environmental variables, that may include factors such as dissolved oxygen, some aspect of water movement (delivering suspended particle food and removing excretion products), and water temperature. The results provide information about which environmental variables contribute most to the genetic structure of populations of the four species, how connectivity amongst VMEs is influenced by environmental variation, if environmental variation plays a consistent role across multiple taxa in explaining population genetic structure, and how such information may be used to inform appropriate management measures, which in the present context, are likely to involve the establishment of a new offshore network of marine protected areas.

## Results

Bivariate correlations revealed that several of the 26 topographic, physico-chemical and biological variables were significantly correlated (Table S1). Notably, pairwise combinations of nitrate, phosphate, aragonite and calcite concentrations were all highly correlated (*r*^2^ > 0.95), as were bottom temperature and temperature-pressure residual (*r*^2^ > 0.95). Therefore, aragonite was selected to represent nitrate, phosphate and calcite, and bottom temperature was selected to represent temperature-pressure residual in the modelling analyses. This selection procedure resulted in 22 variables for the sponge environmental data set (Table 1), and 21 variables for the coral environmental data set (Table 2).

**Table 1:** The coefficients of variables included in general linear models based on individual-based genetic variation for the deep-sea demosponge, *Poecillastra laminaris*, as a function of marker type.

Variable	*COI*	*Cytb*	Microsatellites	
			All loci	Neutral loci
(Intercept)	0.632	0.011	7.583	0.727
botspd	0.071*	—	—	−14.250
bpi.broad	0.000	—	0.003	0.002 *
bpi.fine	0.000*	0.000*	—	—
cdom	—	0.003	—	—
diso2	−0.007*	0.001	2.771	2.156*
disorg	—	—	−82.521	−46.100
dynoc	—	—	—	−4.688
pocc	0.001*	0.000	0.484	—
omega.ara	0.013*	—	—	
seamount	0.003*	—	—	−1.134
sigma.theta	—	—	—	—
slope.percent	0.000*	—	—	−0.112
slopec	—	0.000*	—	0.199
sst	—	−0.488*	—	—
sstgrd	—	0.609	—	—
stdev.slope	—	0.001	—	—
tempbot	—	−0.001	1.752*	0.940*
tidcurr	−0.014	—	—	—
vgpm	0.000*	—	—	0.011
woasalc	−0.017*	—	—	—
woatempc	—	—	−0.529	−0.541
woasilc	—	—	—	—
Multiple R-square	0.508	0.559	0.213	0.231
Adjusted R-square	0.357	0.454	0.128	0.059
p-value	0.003	< 0.001	0.031	0.232

Significance levels at p < 0.05 are labelled as *.

Variables with “—” were not included in the model.

**Table 2:** The coefficients of variables included in general linear models based on individual-based genetic variation for three deep-sea coral species as a function of marker type.

Variable	*Goniocorella dumosa*				*Madrepora oculata*			*Solenosmilia variabilis*			
	*D-loop*	*ITS*	Microsatellites		*ITS*	Microsatellites		*D-loop*	*ITS*	Microsatellites	
			All loci	Neutral loci		All loci	Neutral loci			All loci	Neutral loci
(Intercept)	−6.315	−0.990	31.461	−109.893	0.504	−199.900	−229.200	−0.001	6.655	372.999	223.800
botspd	−0.432*				−0.375			−0.008*			
bpi.broad	< 0.001				0.000*						
bpi.fine	0.001					−0.009*					
cdom	−0.041*		−1.203		0.106*	−5.302*			0.027		
diso2		0.011*			−0.181*		3.179*				
disorg	−0.630		41.871*		−6.072*		−98.730*				
dynoc	−0.629	0.075*	17.758*	8.085*					−0.179*		
pocc	−0.003				−0.021*					−0.139*	
omega.ara	0.108			2.369*		−6.610*		0.002*	−0.240*		
seamount								< 0.001			
sigma.theta	0.208			3.769*		−5.922*			−0.199*		
slope.percent	−0.002*	< 0.001						0.000*			
slopec		0.001		−0.088			−0.214*				
sst	−1.944*	−0.424		−79.505*	−11.080*	−154.400*	−56.200*				
sstgrd	6.949*	0.874*		193.665*	25.630*						
stdev.slope	−0.010				0.016				0.006		
tempbot	0.046				−0.109*	−1.710*				1.437*	1.227*
tidcurr				−1.835*		10.720*		0.004*		8.234*	4.122*
vgpm	< 0.001*				0.001*	0.022*	0.014*				−0.003*
woasalc		0.027				12.110*	6.611*			−9.496*	−5.829*
woatempc		−0.003			0.060		−0.722*				
Multiple R-square	0.568	0.229	0.085	0.149	0.740	0.297	0.141	0.177	0.094	0.062	0.080
Adjusted R-square	0.079	0.106	0.059	0.090	0.583	0.220	0.068	0.106	0.044	0.043	0.062
p-value	0.603	0.089	0.025	0.020	0.001	< 0.001	0.074	0.041	0.110	0.011	0.002

Significance levels at p < 0.05 are labelled as *.

Variables with “—” were not included in the model.

### Model building for individual-based variation

For all 4 species using the different genetic markers a range of models was successfully built. However, because BIC modelling was less successful at building models (i.e. microsatellites in *G. dumosa*) than AIC modelling, only AIC-based models were employed to identify environmental variables that explained genetic variation.

### GLM analyses – sponge

The best model fit (p = 0.003) for individual-based *P. laminaris* explaining variation in the *COI* sequence data contained 11 of the 22 variables, the best model (p < 0.001) for *Cytb* variation contained 9 variables, and the best model fit for variation in the microsatellite data contained 6 and 11 variables for all (p = 0.031) and neutral (p = 0.232) loci, respectively (Table 1). Based on adjusted *r*^2^ values and their associated p-values, the models for the mtDNA sequence variation were a better fit than the models for all loci or neutral loci microsatellite variation (Table 1). Dissolved oxygen was the only variable that occurred in all four models, although it was only statistically significant in two of them (Table 1). Three environmental variables occurred in three of four models, but none were statistically significant on all three occurrences (Table 1). Scatterplots indicated that dissolved oxygen concentration was positively correlated with genetic differentiation for all genetic marker types (p < 0.05) (Figure S1).

### GLM analyses - corals

In total, eleven models were developed and tested for individual-based genetic variation of the three corals (Table 2). Although most multiple *r*^2^ values of the models were low ( < 0.500), seven of 11 models were statistically significant (p < 0.05). For all 11 models, no one variable was significant in explaining genetic differentiation amongst individuals of all three corals. However, seven of the 21 variables were included in five models, and one variable, sea surface temperature, was included in six models.

For *G. dumosa*, two of four models were statistically significant and both involved the microsatellite loci (Table 2). The best model fit for the *D-loop* sequence data included 15 variables (of which six were statistically significant, p < 0.05), but the whole model was not significant (p = 0.603). For the *ITS* sequence data the best model fit contained 8 variables (of which only 3 were statistically significant, p < 0.05), whilst for microsatellite variation the best model fit contained 3 and 7 variables for all and neutral loci, respectively. Amongst all 4 models, dynamic topography was always included and contributed significantly in three, whilst sea surface temperature gradient) was also a significant contributor to all models except for that based on the all loci microsatellite data (Table 2). Scatterplots indicated that dynamic topography was positively correlated with genetic differentiation for all markers except the *D-loop* (p < 0.05) (Figure S2).

For *M. oculata*, two of three models were statistically significant (Table 2). For the *ITS* sequence data, the best model fit contained 12 of the 21 environmental variables, of which 9 variables were significant (p < 0.05) (Table 2). The best model fits for the microsatellite variation included nine variables (all significant) for all loci and seven (all significant) for the neutral loci. Sea surface temperature and surface water primary productivity were present in all models (Table 2). Scatterplots revealed that surface water primary productivity was positively correlated with genetic differentiation, whereas sea surface temperature was negatively correlated with genetic differentiation in two of three models (Figure S3).

For *S. variabilis*, three of four models were statistically significant (Table 2). For *D-loop*, the best model fit contained five variables (three were significant), and a five-variable model (three were significant) also provided the best fit (but not statistically significant) for variation of the *ITS* sequence. The models for microsatellite variation included four variables (all significant) for both the all loci and neutral loci datasets. There were no variables that were shared amongst all four models, but all models except *ITS* included tidal current speed. A positive relationship was observed between tidal current speed and genetic distance, even in the non-significant *ITS* model (Figure S4).

### Hierarchical spatial testing

#### North-Central-South regional populations

Marginal tests resulting from DistLM analysis of population genetic variation at the regional scale showed that the genetic structure of the four species was related to different environmental factors (Table S2). After removal of the variables that did not explain genetic variation, through sequential testing, only one variable was retained in each model. Physico-chemical variables, silicate, bottom current speed, and bottom water temperature) explained variation in the all microsatellites, neutral microsatellites, *COI* sequences and *Cytb* sequences of *P. laminaris*, respectively (Table S2). Bottom water temperature for *P. laminaris* and *M. oculata* and dynamic topography for *M. oculata* and *S. variabilis* had a relative separation amongst genetically-structured regional populations (Figure S5).

For *G. dumosa*, the biological factor surface water primary productivity explained regional genetic structure (r^2^ > 0.669) in both the all and neutral microsatellite loci data sets. For *M. oculata*, dynamic topography and bottom water temperature contributed to genetic differentiation in the all and neutral loci data sets, respectively, whilst salinity was the best interpreter for the genetic structure revealed from *ITS* sequences. For *S. variabilis*, the variable dynamic topography and dissolved oxygen explained variation in the all loci data set, whereas dynamic topography explained most variation in the neutral loci data set (Table S2).

### Geomorphic feature populations

**Figure 2:**
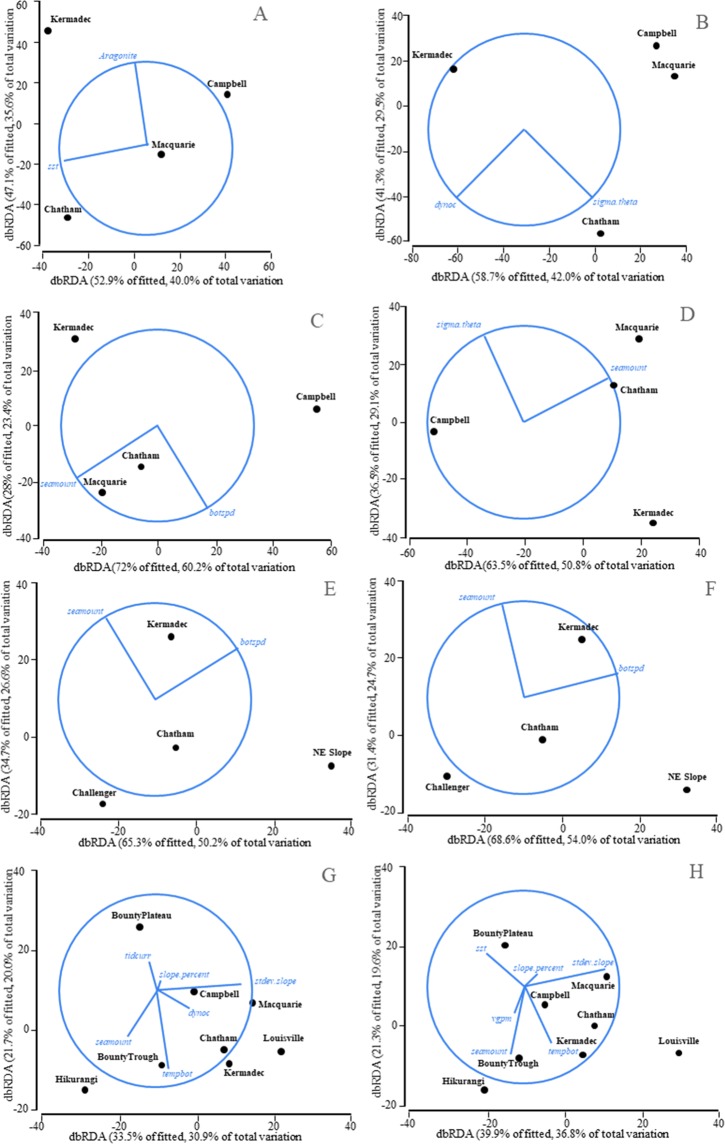
dbRDA plots of genetic variation for *COI* (**A**), *CytB* (**B**), all microsatellites (**D–F**) and neutral microsatellites (**C,E,F**) of *Goniocorella dumosa* (**E,F**), *Solenosmilia variabilis* (**G**,**H**) and *Poecillastra laminaris* (**A–D**). Key to geomorphic feature abbreviated names: NE Slope = NE continental slope, Campbell = Campbell Plateau, Challenger = Challenger Plateau, Chatham = Chatham Rise, Hikurangi = Hikurangi Margin, Kermadec = Kermadec Ridge, Louisville = Louisville Seamount Chain, Macquarie = Macquarie Ridge and Tasman Basin.

Marginal tests resulting from DistLM analyses showed that genetic variation of the sponge *P. laminaris* exhibited a significant relationship with aragonite, bottom current speed, water density, dynamic topography, seamount, sea surface temperature. When considered alone, seamount explained 46.0% of the variability and water density 33.9% in the all loci data set, whilst seamount 49. 9% and bottom current speed 33.6% in the neutral loci data set. In the sequences data set, aragonite and sea surface temperature explained 39.9% and 35.7% in *COI*, and water density and dynamic topography were 41.6% and 29.9% in *Cytb* (Table S3). The dbRDA results illustrated that the populations from the Campbell Plateau and the Kermadec Ridge were genetically different from all other populations, and that water density and seamount were the main contributors to this pattern in the two data sets (Fig. 2A–D). The bottom current speed was in the best explained model in both BEST analyses of *COI* and *Cytb*, whilst salinity and seamount were the common variables in all and neutral microsatellites (Table 3).

**Table 3:** Summary of results of BEST analyses testing for the contribution of all variables to explaining variation in allele frequencies at the geomorphic features level.

Species	Marker	Number of variables	Correlation	BEST model variable selections
*Poecillastra laminaris*	All microsatellite loci	3	1.000	woasalc, tempbot, seamount
	Neutral microsatellite loci	2	1.000	woasalc, seamount
	*COI*	2	0.870	botspd, sstgrd
	*Cytb*	1	0.971	botspd
*Goniocorella dumosa*	All microsatellite loci	1	1.000	omega.ara
	Neutral microsatellite loci	1	0.771	sst
*Solenosmilia variabilis*	All microsatellite loci	4	0.606	bpi.board, sigma.theta, botspd, tempbot
	Neutral microsatellite loci	3	0.600	bpi.board, botspd, tempbot

For *G. dumosa*, the DistLM analysis from the all loci and neutral loci data sets showed that bottom current speed and seamount were the main factors that explained microsatellite variation: they explained 76.9% of the all microsatellites genetic variation and 78.7% of the neutral loci genetic variation (Table S3). The dbRDA plot showed that populations were separated from each other by the combination of bottom current speed and seamount (Fig. 2E,F). Additionally, aragonite was in the best explained model in BEST analyses of all microsatellites, whilst it was sea surface temperature in neutral microsatellites (Table 3).

Microsatellite variation of all loci of *S. variabilis* was partially explained by standard deviation of slope, seamount, bottom water temperature, slope percentage, dynamic topography, and tidal current speed. These six variables explained 92.1% of the observed genetic variability, with the highest contribution being made by seamount (Table S3). In the dbRDA plot, the Louisville Seamount Chain population was separated by standard deviation of slope from the other populations. In the results of the neutral microsatellite loci analysis, standard deviation of slope, seamount, bottom water temperature, sea surface temperature, slope percentage and surface water primary productivity were the factors selected in the DistLM analysis, and these 6 variables accounted for 92.2% of the genetic variation. Amongst these variables, standard deviation of slope was the most significant variable in the DistLM analysis, and it explained 25.9% of the microsatellite genetic variation (Fig. 2). The dbRDA plots revealed that the 8 geomorphic feature populations were genetically different from one another, and that this separation was influenced by the combination of these 6 environmental variables. Furthermore, the Louisville Seamount Chain population was also distinct from the other populations due to the influence of standard deviation of slope (Fig. 2G,H). The BEST analysis of the all and neutral microsatellite data sets agreed that bpi-broad, bottom current speed and bottom water temperature were the variables that explained the genetic variation.

In summary, based on the frequency of occurrences in different models, bottom current speed and bottom water temperature were the most frequently included variables in the models of all examined species.

## Discussion

Seascape genetics as a discipline has advanced dramatically over the last decade as environmental data sets have increased in size and coverage (both spatial and temporal), and as new molecular markers and analytical tools have been developed (reviewed by^32–34^). However, such work has been exclusively focused on coastal and/or shallow water) species and systems, sometimes from the perspective of environmental or evolutionary biology, and sometimes with a management (e.g., fisheries, conservation) focus (e.g.,^38,51–55^). This study is amongst the first to apply the approach to deep-sea species and environments. It proved successful at uncovering relationships between genetic structure and environmental factors, although also revealed some issues that will need to be addressed in future applications of the method.

### Limitations of the present study

Contrasting patterns of population genetic structure have been reported for the sponge and the three coral taxa throughout their distributional ranges in the New Zealand region^3,14^. Here, multi-factorial environmental variability has been examined to help explain the taxon-specific genetic variation. Small sample size is, however, a very big challenge that deep-sea researchers have to face^2,56^, and is a consideration in the interpretation of results from the present study. Because our sampling was based on availability of archived material collected by different research voyages over the years, rather than from a directed sampling programme, the distributions of the specimens analysed here had a spatial bias, with the highest density being from the Chatham Rise, associated with opportunistic collections during fisheries research and benthic biodiversity surveys. This bias may contribute to a failure to reveal seascape genetic patterns at a geomorphic feature level because large samples sizes for the Chatham Rise region may obscure a signal from small sample sizes from other regions. Moreover, because this is the first test of the role of environmental variability explaining genetic variability for deep-sea species, published information from previous studies for comparative purposes was absent. Nonetheless, despite small population sample sizes in some cases (minimum n is 4) and a lack of comparative information about the relationships between environmental factors and genetic differentiation, the results generated here contribute to a more comprehensive understanding about environmental and genetic interactions at both small and large spatial scales.

### Different results between genetic markers

The selected environmental variables varied amongst the different models for each species according to the different genetic markers. For DNA sequence markers, mitochondrial markers had a better performance than nuclear markers, as judged by higher *r*^2^ values in GLM. One possible reason for this result is that the mitochondrial genome is more sensitive than the nuclear genome to environmental variability, perhaps because almost all of the mitogenome is coding whereas most of the nuclear genome is non-coding. Microsatellites, which had a better performance in population genetic studies, were less sensitive to environmental factors, as might be expected for markers that are assumed to be selectively neutral. Whether there is a direct selection-based association between genetic and environmental variation, or whether the environmental variables simply act as barriers to gene flow (a more neutral rather than selection-based explanation) remains to be determined.

Of note is the fact that a single environmental variable may be included in all models for a single species, but for one marker the variable’s contribution was positive and for another marker within the same species the variable’s contribution was negative (see Tables 1 and 2). Determining how a variable may have both a positive and negative contribution to genetic variation within the same species but for different markers will require further investigation. It may, however, be explained by direct selection pressure on one marker (e.g., the mitogenome) and an absence of selection pressure on another marker type (e.g., the nuclear genome). Given the linked nature of regions (genes) on the circular mitogenome, associations (either positive or negative) may be expected to be the same for two or more regions of mitochondrial DNA. The results here do not however, always support this expectation, indicating that different regions of the mitogenome may be responding differentially to the environmental variation, either as settled adults or as dispersive larvae.

### Environmental effects and genetic variation

Topographic factors (such as bathymetric variation), physico-chemical factors (such as temperature, current speed, oxygen, aragonite saturation state, and nutrients), and biological factors (such as surface water Chl-a concentration) were examined directly in the present study. Such variables have previously been identified as environmental drivers for controlling the large-scale distribution of deep-sea corals and sponges^9,20,57–60^. In addition, variables such as hydrodynamics (in various forms), temperature (in various forms), bathymetry, and Chl-a concentration are often reported as being significant explanators of genetic variation in a range of marine taxa, including macroalgae, invertebrates and vertebrates (e.g.^34,38,52,53,55^,).

At the North-Central-South regional scale, the main variables driving the genetic variation amongst coral populations were bottom water temperature, dissolved oxygen, dynamic topography, salinity and surface water primary productivity. Dynamic topography, which is a measure of relative sea surface current velocity, reflects large scale current movements^61^ and may therefore also reflect the dispersal of coral larvae. Significant contributions of dynamic topography to explain genetic variation in *M. oculata* and *S. variabilis* supported the hypothesis in a previous study that large-scale currents associated with the Tropical and Subtropical fronts shaped the regional genetic structure^3^. For *G. dumosa*, the regional structure was influenced by surface water primary productivity revealed in both all and neutral microsatellites, which is related to the amount of food available to suspension-feeding corals^62^. The importance of this variable is also probably related to the influence of currents and fronts on genetic connectivity between the Central region and the North and South regions. That is, *G. dumasa* was more common in relatively shallower water at 400–500 m (compared to *M. oculata* and *S. variabilis*) on the Chatham Rise of the Central region, which is an area of particularly high sea surface primary productivity coincident with the subtropical front (ref for coincidence of high prod and STF). Furthermore, the variables associated with bottom currents and fronts, such as bottom water temperature, dissolved oxygen and salinity, also support the contention that environmental factors associated with the Chatham Rise are responsible for patterns of genetic variation across the three regions^3^. In the sponge, *P. laminaris*, bottom current speed, bottom water temperature and silicate explained more than half of the regional variability in different models. The contribution of silicate to the regional structure was revealed by all microsatellites, whilst bottom current speed and bottom water temperature explained the regional structure detected by *COI*, *Cytb* and neutral microsatellites. This result might indicate that the genome of *P. laminaris* is under some sort of (unknown) selection pressure for silicate, and that the bottom current speed is acting as a genetic barrier by affecting larval settlement and/or the available food source. However, this suggestion will require further evidence to test it. Overall, bottom current was an important factor that contributed to the formation of genetic structures for deep-sea corals and a sponge around New Zealand, and is a variable that is likely to influence genetic variation on multiple spatial scales.

At the geomorphic feature level, the factors that affected genetic connectivity patterns varied across the different species, but seamount was included in the all and neutral microsatellite data sets of all examined species. This indicates that the topography, and associated hydrodynamics, of different geomorphic features may affect the genetic structure of these species - for example, providing an explanation for genetic isolation of populations from Kermadec Ridge, Louisville Seamount Chain and Macquarie Ridge, where topographically-forced oceanographic features such as Taylor columns on the seamounts or along ridge currents could potentially influence larval retention on these features^63^. Previous studies of corals have detected topographically associated genetic patterns: genetic differentiation was significantly different between slope and ridges in *Desmophyllum dianthus*^5^, and in the coral genus *Narella*^11^. However, neither of these studies discussed the relationship between genetic differentiation and topography: further studies are required to verify this relationship. Bottom current speed was another factor that shaped genetic structure in *G. dumosa*, and the terrain variables (e.g., standard deviation of slope and slope percentage) were the most important factors that influenced the genetic variation of populations at the geomorphic features scale, in both the all and neutral microsatellite data sets of *S. variabilis*. For *P. laminaris*, water density (also referred to as sigma-theta) was the crucial explanator of population genetic variation at the geomorphic features scale. This variable reflects the temperature, salinity, and pressure of the water above the geomorphic features, and as such is probably operating as a single proxy variable for the influence of these water mass characteristics on genetic variation. A possible interpretation for all of the aforementioned variables acting as barriers to population connectivity relates to them reflecting the particular environment suitable for larval settlement and development amongst different geomorphic features. That is, the population genetic variation may be driven by different conditions amongst geomorphic features that challenged the larvae from other populations to survive, colonise and/or reproduce once established as an adult, such that little or no genetic contribution amongst local gene pools induced genetic differentiation between local populations. However, to our knowledge, there are no studies available for deep-sea species to provide evidence to support this suggestion.

At the individual level, the variables that elucidated the patterns of genetic variation differed not only amongst species but also by genetic marker. The important variables identified at this level were similar to those identified by habitat suitability modelling^58,61,64,65^, but the importance of each variable in the present study was different from those identified by these previous studies. Sea surface temperature gradient was significant as an explanator of genetic variation in *G. dumosa* and *M. oculata* and sea surface temperature was also significant in most models for these species. Sea surface temperature and sea surface temperature gradient are both related to primary productivity, and the latter to the location of frontal systems^66^. Primary productivity and frontal systems are proxies for food availability^67,68^, and the genetic variation patterns of these two species might therefore be driven by food source availability even at small spatial scales. In *S. variabilis*, although no common variables were found in all four models, tidal current speed was included in three models but not in *ITS*. Tidal current speed is related to the distribution of sedentary suspension-feeding organisms, such as corals and sponges, that rely on currents to deliver food^28,58^. Overall, at small scales, the genetic variations were explained by food-related variables. Food is closely related to an organism’s growth, fecundity, larval dispersal and settlement^69–74^, and thus food availability indirectly effects the genetic contribution made by one population to another population (by fecundity) and/or the genetic connectivity between populations (by larval dispersal and settlement). However, the mechanism by which food resources manipulate genetic connectivity patterns is unclear, and still needs investigation. Interestingly, dynamic topography was an important contribution to explaining the individual-based genetic variation of *G. dumosa* in all 4 models but not at large scales (regional and geomorphic feature level) for this species (it was important at these scales for the other two coral species). As noted above, dynamic topography is related to large-scale current dynamics and can influence large-scale patterns of genetic connectivity via larval dispersal. However, this variable has also been shown previously to be related to the distribution of suspension-feeding organisms through influencing food source availability and variability^28,58^, which provides more support for the explanation that food availability might explain the local genetic structure observed in *G. dumosa*.

For the sponge, *P. laminaris*, dissolved oxygen was the only variable that was included in all four models. Dissolved oxygen is directly related to metabolic rate and low concentrations of dissolved oxygen are expected to adversely affect sponge physiological performance^75^. In addition to dissolved oxygen, POC was also an important variable for *P. laminaris* in the GLM analysis. A significant relationship between POC and genetic structure was also detected in a shallow-sea reef sponge (*Stylissa carteri*)^76^. A previous study has found that POC is a very important food source for sponges^77^, further suggesting that food source availability might be the reason for the genetic differentiation observed in these deep-sea organisms even at small spatial scales. However, in contrast to the three corals, sea surface temperature and sea surface temperature gradient were not main contributors to the local genetic variations in *P. laminaris*. Similarly, sea surface temperature did not contribute to the habitat suitability model for predicting the distribution of the sponge *Geodia*^78^. This result possibly indicates that forces that influence the availability of the food sources and that shape the genetic structure may vary between deep-sea corals and sponges.

Generally, the results at the different spatial scales of this study suggest that bottom currents, food resource and terrain variability shape the genetic structure of both corals and sponges. A previous study has found that deep-sea corals and sponges have similar environmental demands^79^, and this might help to explain the similar genetic differentiations that were observed across these species. However, the variables that influenced the patterns of genetic structure at the different scales were species-specific, a finding that has been reported previously in the limited number of shallow water multi-species studies (reviewed by^34,36^). Nevertheless, while our seascape genetics approach has revealed which environmental variables are potentially important, how these different variables modified the species-specific patterns of genetic connectivity is still unclear. Additionally, high correlation coefficients leave as yet unresolved the question of whether species-specific genetic variation is correlated with a single environmental variable or multiple variables. Investigations of genotypic, phenotypic and environmental variability, as well as of gene-environment interactions are required to resolve these questions.

### Implications for management

Marine environmental managers are increasingly incorporating genetic information into management planning^80–82^, and the results of seascape genetic modelling can help inform management options that conserve genetic structure and connectivity. To maintain population-level genetic diversity and effective population size, it is essential to understand the patterns of genetic connectivity that exist amongst deep-sea populations. However, open spatial population structures, logistical sampling difficulties and unknown barriers to gene flow present challenges to connectivity research. To overcome these challenges, seascape genetics research may open another window to improve understanding of linkages between genetics and the spatial ecology of populations^83^.

The genetic separation of some deep-sea coral and sponge populations into regions or locations that are characterised by particular temperature or food availability regimes illustrates the importance of protecting these VME indicator taxa via spatial management options that include sites (e.g. marine protected areas) that represent these different environmental regimes. For example, these different environmental regimes could be represented by a spatial classification of variables (e.g.,^84,85^) identified by this study as being correlated with genetic variation, which could then be used, along with other data inputs that represent other forms of biological variation in a region, to select the sites of the marine protected areas (e.g.,^2,86^). Additionally, the identification of variables that help to explain coral and sponge genetic variation indicates that networks of protected areas should include representatives of different geomorphic features, and conservation priorities could be allocated to sites that contain particularly complex topography (e.g., seamounts^61,63^).

As seascape genetics moves beyond the search for patterns amongst populations of a single species to a more rigorous approach involving hypothesis testing, individual-based and population-level assessments, greater awareness of spatial and temporal coverage, and comparisons across multiple species, it is expected that new insights into the environmental factors influencing gene flow will be forthcoming, and that such information will be of value in evolutionary studies and management (e.g.,^31,87–89^).

## Materials and methods

### Sample collection

Individuals of the demosponge, *Poecillastra laminaris*, and the stony corals, *Goniocorella dumosa*, *Madrepora oculata* and *Solenosmilia variabilis*, were obtained from the New Zealand National Institute of Water and Atmospheric Research (NIWA) invertebrate collection (NIC). Material had been collected from multiple sites throughout the South Pacific region over multiple years dating back to the 1960s^3,14^. Here, we recognise 12 distinct geomorphic features from which samples had been collected (Table S4). The majority of specimens were from seamount and slope habitats (122–1,805 m water depth). Most material was stored in ethanol, but a small number of samples (mostly sponges) had been air-dried. Only one fragment per species was subsampled from each collection sample (defined as a single sampling event at a single location) to avoid colonies produced by asexual reproduction and/or fragments of the same colony produced by damage caused by sampling gear. To permit testing of spatially explicit data, all specimens were allocated into three hierarchical geographic groups to define the populations for analysis: northern and southern bioprovinces (separated at ~45°S, following^42^), north-central-south regions (separated at ~42°S and ~45°S, following^3,14^) and geomorphic features (12 distinct features - NE continental slope, Bollons Seamount, Bounty Plateau, Bounty Trough, Campbell Plateau, Challenger Plateau, Chatham Rise, Hikurangi Margin, Kermadec Ridge, Louisville Seamount Chain, Macquarie Ridge and Tasman Basin) (Fig. 1). Spatial coverage was dictated by availability of archived material in the NIC. The minimum sample size for a population was set at four to achieve a balance between the validation of results and extracting maximum information content from the specimens. Further details of the ecology and life-histories of the four VME indicator taxa are provided by Zeng *et al*.^3,14^.

### Genetic variation

Two different approaches, sequencing (nucleotide diversity) and genotyping (microsatellite allelic frequencies), were employed to detect species-specific genetic variation. The methodological details of development and identification of appropriate markers and the sequencing and genotyping are described in previous studies^3,14^. Briefly, mitochondrial DNA regions (*COI* and *Cytb*) were sequenced for the sponge, and mitochondrial (*D-loop*) and nuclear (*ITS*) loci were utilised for the corals. In total, 10, 20, 24 and 27 microsatellite loci were genotyped for *P. laminaris*, *G. dumosa*, *M. oculata* and *S. variabilis*, respectively. Because neutral markers can provide unbiased estimates of time since reproductive isolation and the amount of genetic drift^90^, the neutral microsatellite loci identified by LOSITAN^91^ and Micro-checker^92^ were employed for all analyses, where appropriate (Table 4). Following previous studies^41,93^ we also employed all loci, regardless of neutrality, in all analyses where appropriate, because non-neutral loci may be informative about population genetic structure and therefore useful for management purposes. In total, 6, 8, 6 and 12 neutral microsatellite loci were identified for *P. laminaris*, *G. dumosa*, *M. oculata* and *S. variabilis*, respectively. High rates of apparently non-neutral loci are typical of many marine invertebrates, including deep-sea corals and sponges, and may arise for different reasons, including null alleles, coloniality, inbreeding, selection, the Wahlund effect and poor state of sample preservation^2,3,7,14^. Previous work has shown that for population genetic analyses, the two data sets (all loci versus neutral loci only) perform very similarly, but with some small and perhaps important differences^3,14^. The work described here uses the genetic data sets from the population genetics research described by Zeng *et al*.^3,14^, but analyses these in the novel context of environmental variation to test for associations between genetic and environmental variation (pairwise estimates of *Φ*_ST_ and *F*_ST_ are presented in Table S5).

**Table 4:** Number of specimens (in brackets) assayed for each genetic marker type as listed at different spatial scales for four Vulnerable Marine Ecosystem indicator taxa.

Species	Regions	Geomorphic features	Individuals
*Poecillastra laminaris* (Demospongia)	*COI* (n = 53)	*COI* (n = 48)	*COI* (n = 53)
	*Cytb* (n = 51)	*Cytb* (n = 46)	*Cytb* (n = 51)
	Microsatellites (n = 63)	Microsatellites (n = 56)	Microsatellites (n = 63)
*Goniocorella dumosa* (Scleractinia)	Microsatellites (n = 108)	Microsatellites (n = 100)	*ITS* (n = 59)
			*D-loop* (n = 26)
			Microsatellites (n = 108)
*Madrepora oculata* (Scleractinia)	*ITS* (n = 40)	—	*ITS* (n = 33)
	Microsatellites (n = 93)		Microsatellites (n = 93)
*Solenosmilia variabilis* (Scleractinia)	Microsatellites (n = 208)	Microsatellites (n = 200)	*ITS* (n = 96)
			*D-loop* (n = 64)
			Microsatellites (n = 208)

- Test not carried out because of lack of statistically significant population genetic differentiation (Zeng *et al*. 2017).

### Environmental variables

Data for 26 environmental variables − 6 topographic, 17 physico-chemical and 3 biological variables - were obtained from various sources (Table 5). Data for environmental variables were obtained for the same geographic location from which specimens had been sampled. These environmental variables have been shown to potentially influence the distribution of coral and sponge species (see references in Introduction and^61^). The variable calcite was excluded from the analysis of sponge data, whilst silicate was excluded from the analysis of coral data due to their expected lack of biological relevance. Missing values in the environmental data set (about 4.58% missing values) were imputed by Primer-e (v7) using the expectation-maximization algorithm.

**Table 5:** Detailed information of environmental variables included in this study.

Type	Variable	Abbreviation	Unit	Spatial Resolution	Source
Topographic variables	Bathymetric position index – Broad	bpi-broad	—	25 m radius	Wright *et al*. (2005)
	Bathymetric position index – Fine	bpi-fine	—	5 m radius	Wright *et al*. (2005)
	Seamount	seamount	Binomial (yes/no)	1 km	Rowden *et al*. (2008)
	Slope in percent	slope-percent	%	0.00001	Jenness (2012)
	Slope	slopec	—	0.25° × 0.2°	Becker & Sandwell (2008)
	Standard deviation of slope	stdev-slope	—	3 × 3 window	Grohmann *et al*. (2011)
Physico-chemical variables	Sea surface temperature	sst	Degree	1 km	NOAA satellite data
	Sea surface temperature gradient	sstgrd	°C km^−1^	1 km	Uddstrom & Oien (1999)
	Bottom current speed	botspd	m s^–1^	1 km	Hadfield *et al*. (2002)
	Bottom water temperature	tempbot	°C	1 km	CARS (2009) (www.cmar.csiro.au/cars)
	Bottom water temperature residuals	tempres	°C km^–1^	1 km	CARS (2009), Leathwick *et al*. (2012)
	Temperature	woatempc	°C	0.25°	Boyer *et al*. (2005)
	Tidal current speed	tidcurr	m s^−1^	1 km	Walters *et al*. (2001), Hadfield *et al*. (2002)
	Water density	sigma.theta	kg m^−3^	0.01	NIWA
	Dissolved organic matter	disorg	m^−1^	1 km	NASA SeaDas
	Dynamic topography	dynoc	m	1 km	AVISO (http://www.aviso.oceanobs.com)
	Nitrate	woanitc	μmol l^−1^	1°	Garcia *et al*. (2006)
	Aragonite	omega.ara	ΩARAG	—	CARS (2009)
	Calcite	omega.cal	ΩCALC	—	CARS (2009)
	Dissolved oxygen	diso2	ml l^−1^	1°	Garcia *et al*. (2005)
	Phosphate	woaphosc	μmol l^−1^	1°	Garcia *et al*. (2006)
	Salinity	woasalc	PSU	0.25°	Garcia *et al*. (2006)
	Silicate	woasilc	μmol l^−1^	0.25°	Garcia *et al*. (2006)
Biological variables	Chromophoric dissolved organic matter	cdom	aDOM (443) m^–1^	1 km	Pinkerton *et al*. (2006)
	Particulate organic carbon export	pocc	mg Corg.m^−2^ d^−1^	0.08°	Lutz *et al*. (2007)
	Surface water primary productivity	vgpm	mg C m^−2^ d^−1^	1 km	Behrenfield & Falkowski (1997)

Highly correlated environmental variables were identified and eliminated from subsequent analysis to reduce problems that arise for the interpretation of models that include a large number of collinear predictors^94^. Pearson correlation coefficients between all pairs of environmental variables were calculated for those data collected at the sponge and coral sample locations using the R project (v3.2.2,^95^). To overcome colinearity, one of the predicator variables was eliminated from the data set when bivariate correlations were > 0.95^37^.

### Modelling analyses

To test the hypothesis that multi-factorial environmental variation does not significantly explain species-specific genetic variation, three statistical methodologies were employed:

(i) a generalized linear model (GLM), which is a multiple regression analysis between a number of environmental variables and a dependent variable (mean pairwise genetic distances between individuals of any one species). The individual pairwise distances from DNA sequences were generated using MEGA (v6), and a pairwise individual-by-individual genotypic distance matrix was calculated from the microsatellite data using the Codom-Genotypic distance option within GenAlEx^96^. This approach is a modification (based on pairwise individual genetic distance) of the approach using mean pairwise genetic distance between populations employed by^38^ and^37^. GLM analyses were performed separately for each species using a stepwise model, and the best model fit was chosen based on the lowest Akaike Information Criterion (AIC) and Bayesian Information Criterion (BIC) value models. All analyses were conducted using the package MASS in R. Scatterplots of the best-fit model were generated using the package ggplot2 in R.
(ii) a distance-based linear model (DistLM) routine in PERMANOVA+ ^97^ that tests for associations between genetic and environmental variation^37^. DistLM was used to perform an ordination of fitted values from a given model and is constrained to find linear combinations of predictor variables (environmental data) that explain the greatest variation in the data cloud (population-specific haplotypic or allele frequencies). The best-fitting relationship was chosen for the final regression models by comparing the adjusted *r*^2^ selection value. Relationships between environmental parameters were initially examined by analysing each predictor separately (marginal tests), and then sequentially using the adjusted *r*^2^ selection procedure. Similarity matrices in DistLM analyses were built using Bray-Curtis resemblance matrices of haplotypic/allelic frequencies. For each species, haplotypic frequency matrices were calculated for each population in Excel, and allelic frequency matrices were calculated using GenAlEx. The p-values for individual predictor variables were obtained using 9,999 permutations. Distance-based redundancy analysis (dbRDA) plots were also generated using PERMANOVA+ to visualise the results once the best DistLM model of each species was obtained.
(iii) a biological environmental stepwise analysis (BEST) that tests for the relationship between resemblance matrices of dependent (species-specific and population-specific haplotypic/allelic frequencies) and predictor (environmental) variables^38^. The BVSTEP analysis in Primer-e was utilised to search for the best model fit between genetic and environmental variables. The best-fitting relationship was chosen for the final regression models by comparing adjusted *r*^2^ selection criterion. A Bray–Curtis resemblance matrix was employed for the haplotypic/allelic frequency variables and a Euclidean distance resemblance matrix was employed for the environmental variables. To test for species-specific correlations between the two matrices, the BIOENV subroutine of the BEST routine was implemented using the non-parametric Spearman correlation coefficient method (r_S_) to test all combinations of factors.

These three analytical approaches operate in slightly different ways by testing different associations between the species-specific and site-specific genetic and environmental data. GLM is based on genetic variation at the individual, not population level. Whilst DistLM and BEST are based on population-specific haplotypic/allelic frequencies per locus, the former is a permutational model building analysis whereas the latter is a non-parametric test of all possible models, from the 26 single variable models to the one 26-factor model. All three analyses are species-specific and employ the same population-specific environmental data sets.

Rather than testing all possible data set combinations (i.e., all species × all genetic markers × all environmental variables) we only carried out seascape genetics analyses where significant spatial population genetic differentiation has already been identified. This is based on previous studies of deep-sea VME indicator coral^3^ and sponge^14^ population genetic variation. However, the dbRDA and BEST tests do not allow for testing of difference between only two groups of data, which means that testing of differences at the Northern-Southern provinces level could not be carried out. Instead, we focus on analyses of regional and geomorphic population genetic variation. In total, we conducted 15 analyses of individual-based genetic diversity for all four species and their respective markers, plus we conducted a further 12 tests of population-level genetic diversity at the scale of regions and geomorphic features, across all four species and their respective markers (Table 4).

## Supplementary information

Dataset1.

## Data Availability

Data in study are available in the supplementary files.
