# Supplementary material for: Species-specific genetic variation in response to deep-sea environmental variation amongst Vulnerable Marine Ecosystem indicator taxa
Source: Sci Rep. 2020 Feb 18;10:2844. doi: 10.1038/s41598-020-59210-0 (PMC7028729; doi:10.1038/s41598-020-59210-0)
Supplement: Supplementary file 1 — Dataset1. [file 41598_2020_59210_MOESM1_ESM.docx]

**Species-specific genetic variation in response to deep-sea environmental variation amongst Vulnerable Marine Ecosystem indicator taxa**

Cong Zeng^1,2,3,4^, Ashley A. Rowden^2,3^, Malcolm R. Clark^3^, Jonathan P. A. Gardner^2^

1 - College of Animal Science and Technology, Hunan Agricultural University, Changsha, China

2 - School of Biological Sciences, Victoria University of Wellington, Wellington 6140, New Zealand

3 - Coasts and Oceans National Centre, National Institute for Water and Atmospheric Research, Private Bag 14901, Kilbirnie, Wellington, New Zealand

4 – School of Oceanography, Shanghai Jiao Tong University, Shanghai 200030, China

Table S1. Correlation analyses between pairs of environmental variables

|  | bpi.broad | bpi.fine | sigma.theta | slope.percent | stdev.slope | diso2 | pocc | slopec | woanitc | woaphosc | woasalc | woasilc | woatempc | omega.ara | omega.cal | botspd | cdom | disorg | dynoc | sstgrd | sst | tempbot | tempres | tidcurr | vgpm | seamount |
| --- | --- | --- | --- | --- | --- | --- | --- | --- | --- | --- | --- | --- | --- | --- | --- | --- | --- | --- | --- | --- | --- | --- | --- | --- | --- | --- |
| bpi.broad |  |  |  |  |  |  |  |  |  |  |  |  |  |  |  |  |  |  |  |  |  |  |  |  |  |  |
| bpi.fine | 0.753 |  |  |  |  |  |  |  |  |  |  |  |  |  |  |  |  |  |  |  |  |  |  |  |  |  |
| sigma.theta | 0.172 | 0.132 |  |  |  |  |  |  |  |  |  |  |  |  |  |  |  |  |  |  |  |  |  |  |  |  |
| slope.percent | 0.650 | 0.571 | 0.294 |  |  |  |  |  |  |  |  |  |  |  |  |  |  |  |  |  |  |  |  |  |  |  |
| stdev.slope | 0.777 | 0.804 | 0.240 | 0.760 |  |  |  |  |  |  |  |  |  |  |  |  |  |  |  |  |  |  |  |  |  |  |
| diso2 | -0.253 | -0.434 | -0.596 | -0.380 | -0.436 |  |  |  |  |  |  |  |  |  |  |  |  |  |  |  |  |  |  |  |  |  |
| pocc | -0.537 | -0.475 | -0.623 | -0.479 | -0.550 | 0.584 |  |  |  |  |  |  |  |  |  |  |  |  |  |  |  |  |  |  |  |  |
| slopec | 0.576 | 0.627 | 0.232 | 0.695 | 0.639 | -0.377 | -0.432 |  |  |  |  |  |  |  |  |  |  |  |  |  |  |  |  |  |  |  |
| woanitc | 0.280 | 0.316 | 0.838 | 0.327 | 0.376 | -0.797 | -0.729 | 0.305 |  |  |  |  |  |  |  |  |  |  |  |  |  |  |  |  |  |  |
| woaphosc | 0.272 | 0.339 | 0.849 | 0.309 | 0.377 | -0.801 | -0.716 | 0.285 | **0.986** |  |  |  |  |  |  |  |  |  |  |  |  |  |  |  |  |  |
| woasalc | -0.007 | 0.112 | -0.538 | 0.084 | 0.070 | 0.026 | 0.253 | 0.104 | -0.539 | -0.540 |  |  |  |  |  |  |  |  |  |  |  |  |  |  |  |  |
| woasilc | 0.328 | 0.473 | 0.789 | 0.391 | 0.451 | -0.790 | -0.649 | 0.391 | 0.848 | 0.861 | -0.212 |  |  |  |  |  |  |  |  |  |  |  |  |  |  |  |
| woatempc | -0.241 | -0.266 | -0.883 | -0.265 | -0.320 | 0.610 | 0.663 | -0.232 | -0.937 | -0.942 | 0.677 | -0.841 |  |  |  |  |  |  |  |  |  |  |  |  |  |  |
| omega.ara | -0.233 | -0.304 | -0.872 | -0.292 | -0.350 | 0.772 | 0.691 | -0.265 | **-0.960** | **-0.967** | 0.548 | -0.846 | 0.937 |  |  |  |  |  |  |  |  |  |  |  |  |  |
| omega.cal | -0.234 | -0.306 | -0.871 | -0.295 | -0.353 | 0.776 | 0.692 | -0.267 | **-0.960** | **-0.967** | 0.541 | -0.849 | 0.936 | **1.000** |  |  |  |  |  |  |  |  |  |  |  |  |
| botspd | -0.061 | -0.238 | 0.294 | -0.040 | -0.171 | -0.106 | -0.156 | 0.015 | 0.205 | 0.173 | -0.182 | 0.072 | -0.181 | -0.232 | -0.231 |  |  |  |  |  |  |  |  |  |  |  |
| cdom | -0.396 | -0.288 | -0.335 | -0.234 | -0.307 | 0.011 | 0.634 | -0.173 | -0.324 | -0.314 | 0.249 | -0.340 | 0.431 | 0.287 | 0.285 | 0.040 |  |  |  |  |  |  |  |  |  |  |
| disorg | -0.470 | -0.440 | -0.311 | -0.308 | -0.399 | 0.350 | 0.795 | -0.271 | -0.445 | -0.438 | 0.083 | -0.429 | 0.403 | 0.388 | 0.390 | 0.006 | 0.678 |  |  |  |  |  |  |  |  |  |
| dynoc | 0.446 | 0.513 | -0.070 | 0.361 | 0.427 | -0.441 | -0.139 | 0.317 | 0.089 | 0.116 | 0.256 | 0.156 | 0.084 | -0.096 | -0.100 | -0.251 | 0.260 | -0.123 |  |  |  |  |  |  |  |  |
| sstgrd | -0.477 | -0.456 | -0.398 | -0.418 | -0.496 | 0.520 | 0.774 | -0.408 | -0.574 | -0.545 | 0.107 | -0.536 | 0.481 | 0.499 | 0.502 | 0.078 | 0.433 | 0.731 | -0.234 |  |  |  |  |  |  |  |
| sst | -0.547 | -0.472 | -0.463 | -0.410 | -0.518 | 0.477 | 0.749 | -0.405 | -0.605 | -0.579 | 0.242 | -0.548 | 0.548 | 0.542 | 0.544 | 0.062 | 0.461 | 0.664 | -0.212 | 0.942 |  |  |  |  |  |  |
| tempbot | 0.087 | 0.227 | -0.332 | 0.184 | 0.202 | -0.353 | 0.068 | 0.151 | -0.150 | -0.163 | 0.711 | 0.001 | 0.387 | 0.193 | 0.184 | -0.244 | 0.394 | 0.027 | 0.641 | -0.152 | -0.032 |  |  |  |  |  |
| tempres | 0.090 | 0.227 | -0.332 | 0.192 | 0.206 | -0.352 | 0.066 | 0.153 | -0.152 | -0.165 | 0.714 | -0.002 | 0.389 | 0.193 | 0.185 | -0.238 | 0.392 | 0.026 | 0.639 | -0.152 | -0.029 | **0.999** |  |  |  |  |
| tidcurr | -0.391 | -0.347 | -0.441 | -0.496 | -0.488 | 0.589 | 0.786 | -0.418 | -0.583 | -0.549 | 0.057 | -0.440 | 0.423 | 0.520 | 0.522 | -0.261 | 0.343 | 0.537 | -0.157 | 0.659 | 0.573 | -0.131 | -0.138 |  |  |  |
| vgpm | -0.376 | -0.243 | -0.368 | -0.157 | -0.257 | 0.025 | 0.659 | -0.092 | -0.400 | -0.388 | 0.373 | -0.366 | 0.517 | 0.365 | 0.363 | 0.007 | 0.901 | 0.781 | 0.300 | 0.521 | 0.553 | 0.438 | 0.437 | 0.309 |  |  |
| seamount | 0.632 | 0.651 | 0.112 | 0.485 | 0.597 | -0.349 | -0.389 | 0.419 | 0.277 | 0.279 | 0.021 | 0.299 | -0.199 | -0.248 | -0.249 | -0.026 | -0.140 | -0.304 | 0.382 | -0.376 | -0.389 | 0.177 | 0.179 | -0.407 | -0.123 |  |

Note: numbers in bold indicate significant correlation coefficients (p < 0.05).

Table S2. Sequential test results of the DistLM analysis for regional-level genetic variation amongst four deep-sea VME indicator taxa

| Species | Marker | Loci | Variable | Adj R^2^ | SS(trace) | Proportion |
| --- | --- | --- | --- | --- | --- | --- |
| *Poecillastra laminaris* | *COI* | | botspd | 0.090 | 4810.0 | 54.511 |
|  | *Cytb* | | tempbot | 0.246 | 5036.4 | 62.314 |
|  | Microsatellites | All | woasilc | 0.177 | 1868.1 | 58.828 |
|  |  | Neutral | botspd | 0.248 | 1514.3 | 62.401 |
| *Goniocorella dumosa* | Microsatellites | All | vgpm | 0.669 | 2430.0 | 83.447 |
|  |  | Neutral | vgpm | 0.740 | 2410.1 | 86.988 |
| *Madrepora oculata* | *ITS* | | woasalc | 0.282 | 4340.6 | 64.090 |
|  | Microsatellites | All | dynoc | 0.342 | 1406.6 | 67.123 |
|  |  | Neutral | tempbot | 0.320 | 1315.1 | 65.976 |
| *Solenosmilia variabilis* | Microsatellites | All | diso2 | 0.289 | 354.4 | 64.456 |
|  |  | Neutral | dynoc | 0.398 | 361.3 | 69.890 |

Table S3. Sequential test results of the DistLM analysis for microsatellite data amongst geomorphic features for all species

| Species | Loci | Variable | Adj R^2^ | SS (trace) | p | Proportion |
| --- | --- | --- | --- | --- | --- | --- |
| *Goniocorella dumosa* | all microsatellites | botspd | 0.040 | 1332.200 | 0.392 | 35.980 |
|  |  | seamount | 0.306 | 1513.900 | 0.280 | 40.888 |
|  |  | botspd | 0.174 | 1657.800 | 0.199 | 44.936 |
|  |  | seamount | 0.360 | 1244.000 | 0.334 | 33.721 |
| *Solenosmilia variabilis* | all microsatellites | stdev.slope | 0.066 | 1229.600 | 0.104 | 19.992 |
|  |  | seamount | 0.182 | 1327.100 | 0.026 | 21.577 |
|  |  | tempbot | 0.276 | 1047.600 | 0.850 | 17.033 |
|  |  | slope.percent | 0.345 | 819.710 | 0.266 | 13.327 |
|  |  | dynoc | 0.368 | 615.680 | 0.431 | 10.010 |
|  |  | tidcurr | 0.446 | 624.430 | 0.439 | 10.152 |
|  | neutral microsatellites | stdev.slope | 0.136 | 1332.800 | 0.027 | 25.925 |
|  |  | seamount | 0.234 | 994.690 | 0.048 | 19.348 |
|  |  | tempbot | 0.297 | 749.610 | 0.161 | 14.581 |
|  |  | sst | 0.329 | 585.540 | 0.372 | 11.389 |
|  |  | slope.percent | 0.388 | 578.930 | 0.367 | 11.261 |
|  |  | vgpm | 0.454 | 498.290 | 0.459 | 9.692 |
| *Poecillastra laminaris* | all microsatellites | seamount | 0.190 | 3330.700 | 0.166 | 46.010 |
|  |  | sigma.theta | 0.397 | 2452.200 | 0.360 | 33.874 |
|  | neutral microsatellites | seamount | 0.250 | 0.483 | 0.174 | 49.985 |
|  |  | botspd | 0.508 | 2342.200 | 0.363 | 33.613 |
|  | *COI* | sst | 0.099 | 5192.300 | 0.093 | 39.913 |
|  |  | omega.ara | 0.269 | 4647.000 | 0.340 | 35.722 |
|  | *CytB* | sigma.theta | 0.125 | 5390.000 | 0.088 | 41.641 |
|  |  | dynoc | 0.146 | 3867.900 | 0.583 | 29.878 |

Table S4. Geomorphic feature (site), collection date and sample size collection information for the three deep-sea corals and one sponge species.

| Geomorphic feature | Date of collection | *Poecillastra laminaris* | *Goniocorella dumosa* | *Madrepora oculata* | *Solenosmilia variabilis* |
| --- | --- | --- | --- | --- | --- |
| Bollons Seamount | 2003 |  |  | 1 | 2 |
| Bounty Plateau | 1979-2008 |  |  | 4 | 4 |
| Bounty Trough | 2006-2008 |  |  | 2 | 5 |
| Campbell Plateau | 1978-2010 | 4 | 1 | 8 | 8 |
| Challenger Plateau | 1961-2013 | 3 | 3 | 5 | 1 |
| Chatham Rise | 1963-2015 | 19 | 48 | 42 | 71 |
| Hikurangi Margin | 2002-2010 | 2 | 1 | 1 | 4 |
| Kermadec Ridge | 1989-2012 | 11 | 3 | 6 | 33 |
| Louisville Seamount | 2014 |  |  |  | 14 |
| Macquarie Ridge | 1998-2008 | 14 | 2 |  | 13 |
| NE Continental Slope | 2000 |  | 3 |  |  |
| Tasman Basin | 1988-2009 |  |  |  | 3 |

Note: Only the geomorphic features (total sample size ≤ 3) were listed in the table.

Table S5. Pairwise estimates of *Φ*_ST_ and *F*_ST_ derived from DNA sequence and microsatellite analyses of genetic variation of one sponge and three coral species.

Table S5a. Pairwise *Φ_ST_* values for *COI* (below diagonal) and *Cytb* (above diagonal) between populations of the sponge, *Poecillastra laminaris.*

| Populations | Kermadec Ridge | Three Kings Ridge | Challenger Plateau | NW Slope | Hikurangi Margin | Chatham Rise | Campbell Plateau | Bounty Trough | Macquarie Ridge | Antarctica |
| --- | --- | --- | --- | --- | --- | --- | --- | --- | --- | --- |
| Kermadec Ridge |  | - | 0.891** | -1.000 | 0.410 | 0.371** | 0.937** | 0.700* | 0.400** | 0.891 |
| Three Kings Ridge | -0.875 |  | - | - | - | - | - | - | - | - |
| Challenger Plateau | 0.780* | 1.000 |  | 1.000 | 0.500 | -0.058 | 1.000* | 0.421 | 0.432** | 1.000 |
| NW Slope | -0.875 | 0 | 1.000 |  | -1.000 | -0.063 | 1.000 | -0.467 | -0.039 | 1.000 |
| Hikurangi Margin | 0.264 | -1.000 | 0 | -1.000 |  | -0.012 | 0.848 | 0.063 | 0.154 | 0.429 |
| Chatham Rise | 0.329** | -0.101 | -0.104 | -0.101 | -0.072 |  | 0.474** | 0.144 | 0.226** | 0.309 |
| Campbell Plateau | 0.95** | 1.000 | 1.000 | 1.000 | 0.919 | 0.469 |  | 0.250 | 0.434* | 1.000 |
| Bounty Trough | 0.652* | -0.727 | 0.136 | -0.727 | 0 | 0.148* | 0.250 |  | 0.058 | -0.294 |
| Macquarie Ridge | 0.650** | 0.444 | 0.502* | 0.444 | 0.462* | 0.210** | 0.467** | 0.238 |  | 0.064 |
| Antarctica | 0.899 | 1.000 | 1.000 | 1.000 | 0.600 | -0.092 | 1.000 | -1.000 | -0.583 |  |

Significant values of p<0.05 are marked as *, and p<0.01 are marked as **.

- data are unavailable.

Table S5b. Pairwise *F_ST_* values for microsatellite variation between populations of the sponge, *Poecillastra laminaris.*

|  | Campbell Plateau | Chatham Rise | Kermadec Ridge | Macquarie Ridge |
| --- | --- | --- | --- | --- |
| Campbell Plateau |  |  |  |  |
| Chatham Rise | -0.005 |  |  |  |
| Kermadec Ridge | 0.156* | 0.061* |  |  |
| Macquarie Ridge | 0.023 | -0.015 | 0.035 |  |

Table S5c. Pairwise *Φ*_ST_ values for ITS of *Madrepora oculata* (above diagonal) and *Solenosmilia variabilis* (below diagonal) between coral populations.

|  | Bounty Plateau | Campbell Plateau | Chatham Rise | Kermadec Ridge | Louisville Seamount Chain | Macquarie Ridge |
| --- | --- | --- | --- | --- | --- | --- |
| Bounty Plateau |  | 0.063 | 0.148 | 0.447 | - | - |
| Campbell Plateau | - |  | 0.175 | 0.5 | - | - |
| Chatham Rise | - | -0.087 |  | 0.457** | - | - |
| Kermadec Ridge | - | -0.094 | 0.019 |  | - | - |
| Louisville Seamount Chain | - | 0 | -0.014 | -0.018 |  | - |
| Macquarie Ridge | - | -0.018 | 0.032 | 0.223* | 0.100* |  |

Significant values of p < 0.05 are marked as *, and p < 0.01 are marked as **.

Data for *G. dumosa* not included because all *Φ*_ST_ values are NS.

Table S5d. Pairwise *F*_ST_ values for all (below diagonal) and neutral (above diagonal) loci between populations of the coral, *Goniocorella dumosa*.

|  | NE continental slope | Challenger Plateau | Chatham Rise | Kermadec Ridge |
| --- | --- | --- | --- | --- |
| NE continental slope |  | -0.018 | 0.033 | 0.009 |
| Challenger Plateau | -0.047 |  | -0.006 | 0.015 |
| Chatham Rise | 0.001 | -0.014 |  | 0.044* |
| Kermadec Ridge | -0.012 | 0.003 | 0.024* |  |

Table S5e. Pairwise *F*_ST_ values for all (below diagonal) and neutral (above diagonal) loci between populations of the coral, *Solenosmilia variabilis*.

|  | Bounty Plateau | Bounty Trough | Campbell Plateau | Chatham Rise | Hikurangi Margin | Kermadec Ridge | Louisville Seamount Chain | Macquarie Ridge |
| --- | --- | --- | --- | --- | --- | --- | --- | --- |
| Bounty Plateau |  | -0.022 | -0.002 | 0.031* | 0.053 | 0.048* | 0.053 | 0.019 |
| Bounty Trough | -0.037 |  | -0.001 | 0.008 | -0.038 | -0.018 | 0.034 | 0.02 |
| Campbell Plateau | 0.02 | -0.003 |  | 0.061** | 0.034 | 0.086** | 0.115** | 0.061** |
| Chatham Rise | 0.025 | 0.003 | 0.048** |  | 0.033* | 0.022** | 0.043** | -0.009 |
| Hikurangi Margin | 0.094 | 0.02 | 0.052 | 0.048** |  | -0.008 | 0.068* | 0.038* |
| Kermadec Ridge | 0.035 | -0.014 | 0.061** | 0.014** | 0.008 |  | 0.032** | 0.031** |
| Louisville Seamount Chain | 0.06 | 0.029 | 0.085** | 0.035** | 0.081** | 0.027** |  | 0.045** |
| Macquarie Ridge | 0.027 | 0.013 | 0.041** | -0.008 | 0.030* | 0.018** | 0.036** |  |

Significant values of p < 0.05 are marked as *, and p < 0.01 are marked as **.

Data for *M. oculata* not shown because all *F_ST_* values were not statistically significant.


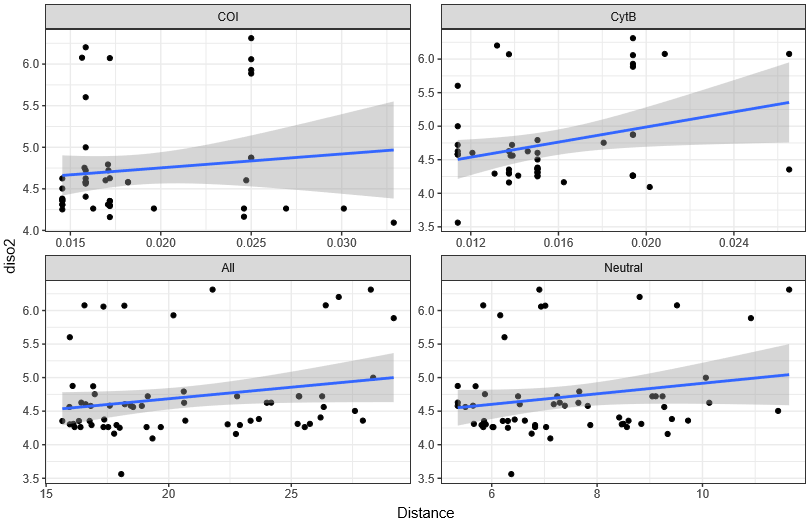


Figure S1. Scatterplot of genetic distance (Distance) as a function of dissolved oxygen (diso2) for the deep-sea sponge *Poecillastra laminaris* across four genetic marker types.

Note: The blue line is the fitted linear regression line and the grey area is the 95% confidence interval.


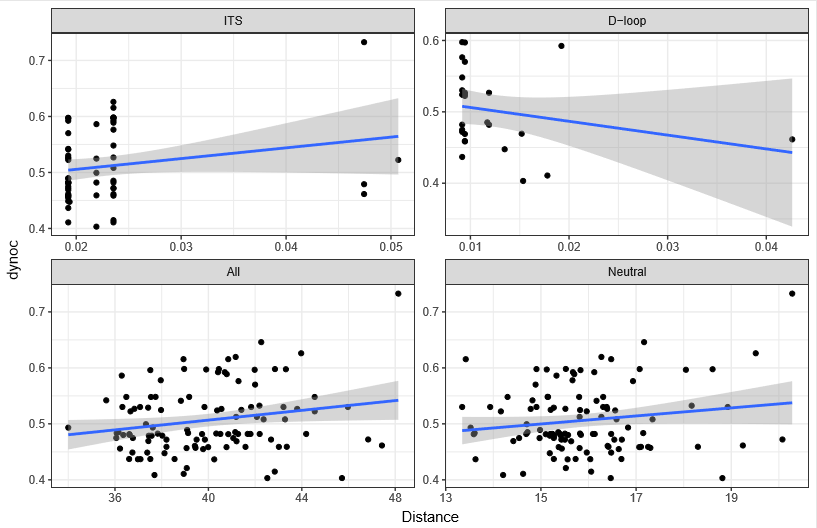


Figure S2. Scatterplot of genetic distance (Distance) as a function of dynamic topography (dynoc) for the deep-sea coral *Goniocorella dumosa* across four genetic marker types.

Note: The blue line is the fitted linear regression line and the grey area is the 95% confidence interval.


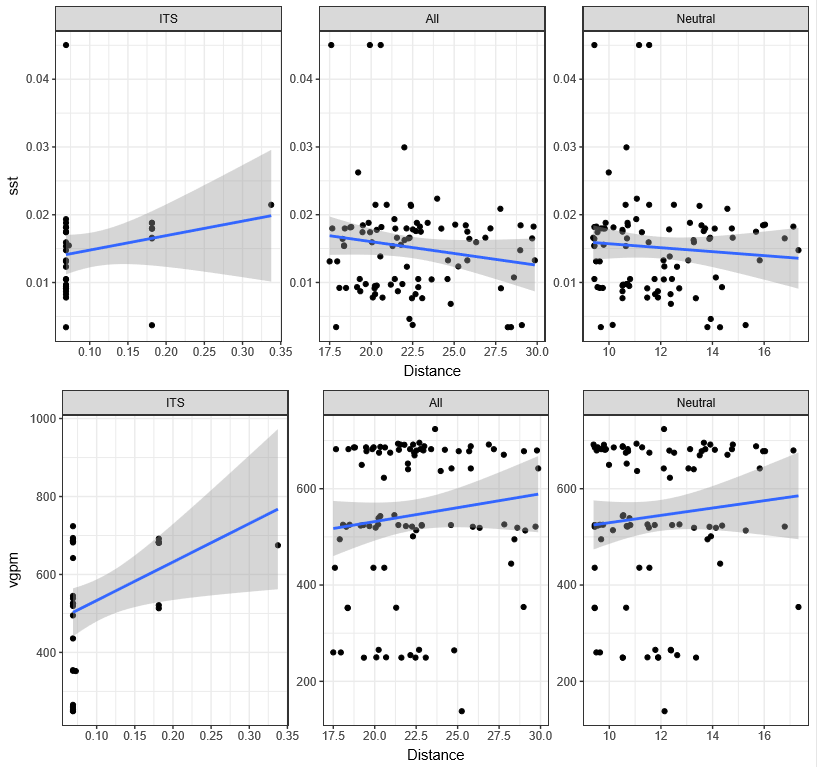


Figure S3. Scatterplot of genetic distance (Distance) as a function of sea surface temperature (sst) and surface water primary productivity (vgpm) for the deep-sea coral *Madrepora oculata* across three genetic marker types.

Note: The blue line is the fitted linear regression line and the grey area is the 95% confidence interval.


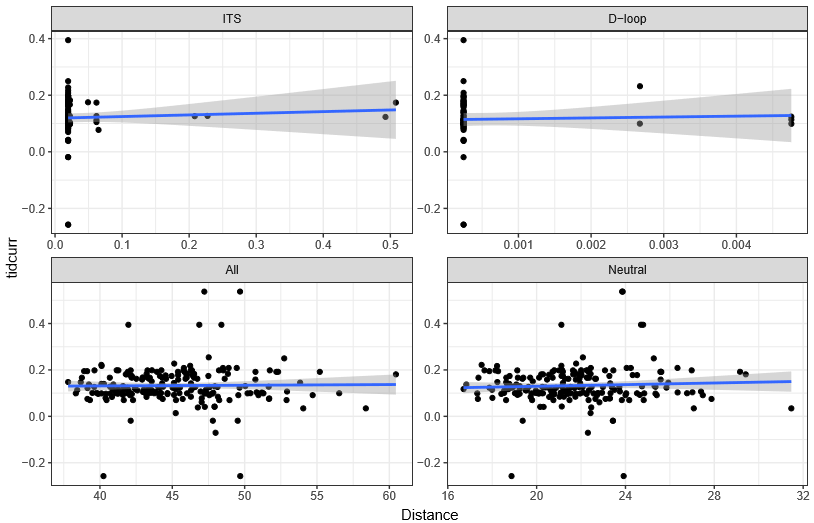


Figure S4. Scatterplot of genetic distance (Distance) as a function of tidal current speed (tidcurr) for the deep-sea coral *Solenosmilia variabilis* across four genetic markers.

Note: The blue line is the fitted linear regression line and the grey area is the 95% confidence interval.


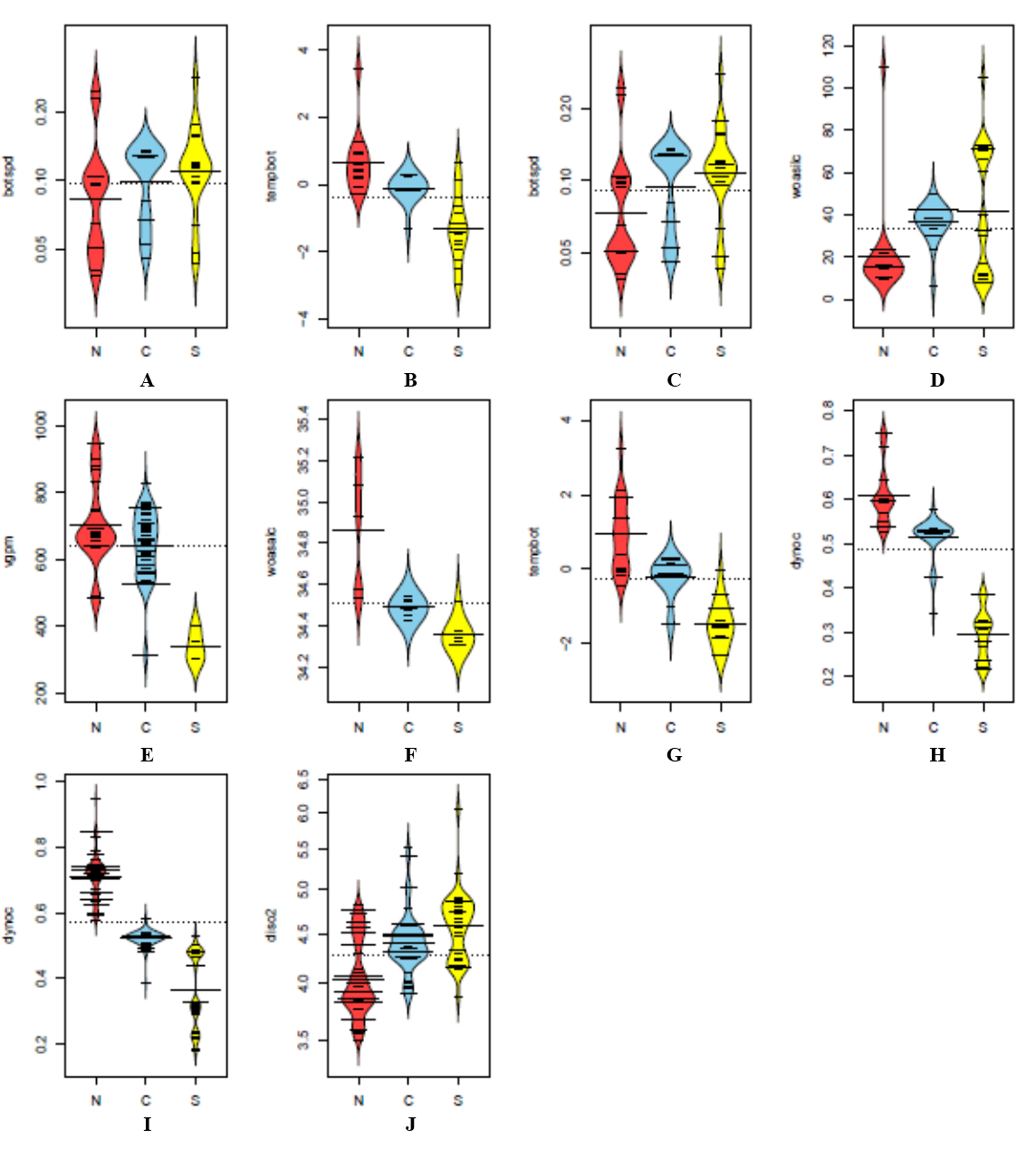


Figure S5. Comparison across north-central-south regional populations of their distribution range along the environmental variables that effected the genetic structures in DistLM analyses amongst *Poecillastra laminaris* (A, B, C, D), *Goniocorella dumosa* (E), *Madrepora oculata* (F, G, H) and *Solenosmilia variabilis* (I, J) and different markers (*COI* (A), *Cytb* (B), *ITS* (F), all microsatellite loci (D, E, H, J) and neutral microsatellite loci (C, E, G, I)). Individual observations are shown as small horizontal white lines within the estimated density trace. Dotted lines represent the median over all subpopulations.
